# Development of Sheep Intestinal Organoids for Studying Deoxynivalenol-Induced Toxicity

**DOI:** 10.3390/ijms26030955

**Published:** 2025-01-23

**Authors:** Hongyu Wang, Xige He, Miaomiao Zhang, Na Fan, Zongxuan Yang, Ting Shen, Jiaojiao Guo, Yongli Song, Guifang Cao, Yongbin Liu, Xihe Li, Buhe Nashun

**Affiliations:** 1 Inner Mongolia Key Laboratory for Molecular Regulation of the Cell, Inner Mongolia University, Hohhot 010070, China; 22408045@mail.imu.edu.cn (H.W.); 131994998@imu.edu.cn (X.H.); 32308061@mail.imu.edu.cn (M.Z.); 22008037@mail.imu.edu.cn (N.F.); 22108042@mail.imu.edu.cn (Z.Y.); 0222122876@mail.imu.edu.cn (T.S.); gijlife@imu.edu.cn (J.G.); ylsong@imu.edu.cn (Y.S.); guifangcao@126.com (G.C.); ybliu@imu.edu.cn (Y.L.); 2 State Key Laboratory of Reproductive Regulation and Breeding of Grassland Livestock, School of Life Sciences, Inner Mongolia University, Hohhot 010040, China; 3 Inner Mongolia Saikexing Institute of Breeding and Reproductive Biotechnology in Domestic Animals, Hohhot 011517, China

**Keywords:** sheep, intestinal organoids, deoxynivalenol (DON), intestinal toxicity, PI3K/AKT/GSK3β/β-catenin signaling

## Abstract

Sheep are an important livestock species whose gastrointestinal tract is essential for overall health. Feed contaminants such as bacterial toxins and mycotoxins severely damage the sheep intestine, yet the mechanisms remain mostly elusive partially due to the lack of physiologically relevant in vitro models. Here, we investigated molecular mechanisms underlying deoxynivalenol (DON)-induced toxicity by developing intestinal organoids from isolated intestinal crypts of Hu sheep. The organoids had a central lumen and monolayer epithelium, and could be continuously passaged, cryopreserved, and resuscitated. Histological and transcriptomic analysis showed that the intestinal organoids recapitulate the cell lineages and gene expression characteristics of the original intestinal tissues. Statistical analysis indicated that DON exposure significantly inhibited organoid formation efficiency, as well as the proliferation and activity of intestinal organoid cells. RNA-seq and Western blotting analysis further revealed that DON exposure induces intestinal toxicity by inhibiting the PI3K/AKT/GSK3β/β-catenin signaling pathway. Our study provides a novel example of organoid application in toxicity studies and reveals the signaling pathway involved in DON-induced toxicity in sheep, which is of great significance for improving mitigation strategies for DON.

## 1. Introduction

The mammalian intestinal tract constitutes the second-largest epithelium in the body [1]. As an absorptive epithelium, the intestine is primarily responsible for the uptake of metabolites and the protection against environmental damage [2]. The intestinal epithelium is organized into millions of crypt–villus units. One unit consists of a villus, a finger-like protrusion of the intestinal wall, surrounded by invaginations called crypts. Within the crypt, continuously dividing stem cells give rise to progenitor cells (or transit-amplifying cells), which rapidly proliferate and eventually differentiate into mature intestinal epithelial cells [3]. Organoids are in vitro three-dimensional (3D) cellular clusters derived exclusively from primary tissues, embryonic stem cells (ESCs), or induced pluripotent stem cells (iPSCs), which are capable of self-renewal and self-organization, and exhibit organ functions similar to those of the original tissues [4]. The unique 3D structure, heterogeneity, and cellular functions are physiologically relevant in disease modeling and drug response prediction, thus serving as a link between cell lines and in vivo models [5]. In 2009, Dutch scientist Hans Clevers successfully cultured organoids derived from mouse intestinal stem cells [6]. Since then, the intestinal organoid in animal research has gained significant attention, and intestinal organoids from animals such as pigs, chickens, dogs, bats, cats, and bovines have been successfully established [7,8,9,10,11,12,13,14,15], providing a great model for investigation of interaction mechanisms between nutrients, toxins, and intestinal epithelial cells [16]. However, there have been few relevant studies on sheep intestinal organoids. Since the intestinal health of sheep is crucial for their growth and development, intestinal organoids potentially offer a physiologically relevant model for studying sheep intestinal function and disease mechanisms.

Mycotoxins are secondary metabolites primarily synthesized by various fungi, which can emerge during grain development, milling, storage, and transportation procedures [17,18]. Deoxynivalenol (DON), produced by Fusarium, is found in many food and feed products. It is highly stable and difficult to eliminate, posing a significant risk to human and animal health [19,20]. Following the ingestion of contaminated feed by animals, the toxin can easily spread and damage multiple organs, including the gastrointestinal tract, liver, kidney, and brain [21,22,23]. Notably, the intestine is one of the main organs that respond to ingested DON [24], and numerous studies have shown that DON alters intestinal nutrient absorption, barrier function, and immune response, and induces intestinal lesions in humans and animals [25,26,27]. Given that humans and animals can be exposed to multiple mycotoxins simultaneously, assessing mycotoxin risk should not only focus on individual toxins’ health hazards but also consider the combined toxic effects of their interactions [28]. Low doses of DON and Nivalenol (NIV) can exhibit synergistic pro-inflammatory effects on intestinal up-regulating the gene expression of pro-inflammatory factors [29]. Additionally, research has shown that co-exposure to DON and other toxins can significantly alter the expression of key genes related to apoptosis, leading to synergistic toxic effects [30]. For instance, combined exposure of intestinal cells to DON and aflatoxin B1 has been found to disrupt the balance of survival and death signals, while co-exposure to DON and T-2 toxins has been associated with increased oxidative stress and inflammatory responses, ultimately resulting in intestinal damage [31]. However, the molecular mechanisms underlying DON-induced sheep intestinal toxicity remain mostly elusive, partially due to the lack of proper in vitro experimental models. Immortalized intestinal epithelial cell lines lack cellular diversity and fail to fully replicate tissue function [32,33], while generation of an animal model is expensive and time-consuming, which limits large-scale application [34]. In contrast, intestinal organoids have been shown to be effective models for studying animal diseases and host–microbe interactions on the epithelial surface [16]. To date, mouse and porcine intestinal organoids have been established and used as infection models [35,36]. Therefore, it is likely that sheep intestinal organoids can be used as an important model for studying DON-induced intestinal toxicity, promoting the elucidation of the underlying molecular mechanisms.

Herein, sheep intestinal crypts were isolated, embedded in Matrigel, and cultured in a complete organoid growth medium. Combined histological and transcriptome analyses showed the successful establishment of sheep intestinal organoids, which were subsequently utilized for simulating DON-induced intestinal toxicity in sheep. DON exposure significantly inhibited cell viability and organoid formation efficiency, and disrupted intestinal barrier function. On the molecular level, DON damaged sheep intestinal organoids by inhibiting the PI3K/AKT/GSK3β/β-catenin signaling pathway. Our findings, therefore, highlighted the effectiveness of intestinal organoids in studying DON-induced feed toxicity in livestock animals.

## 2. Results

### 2.1. Establishment of Sheep Intestinal Organoids

**Figure 1:**
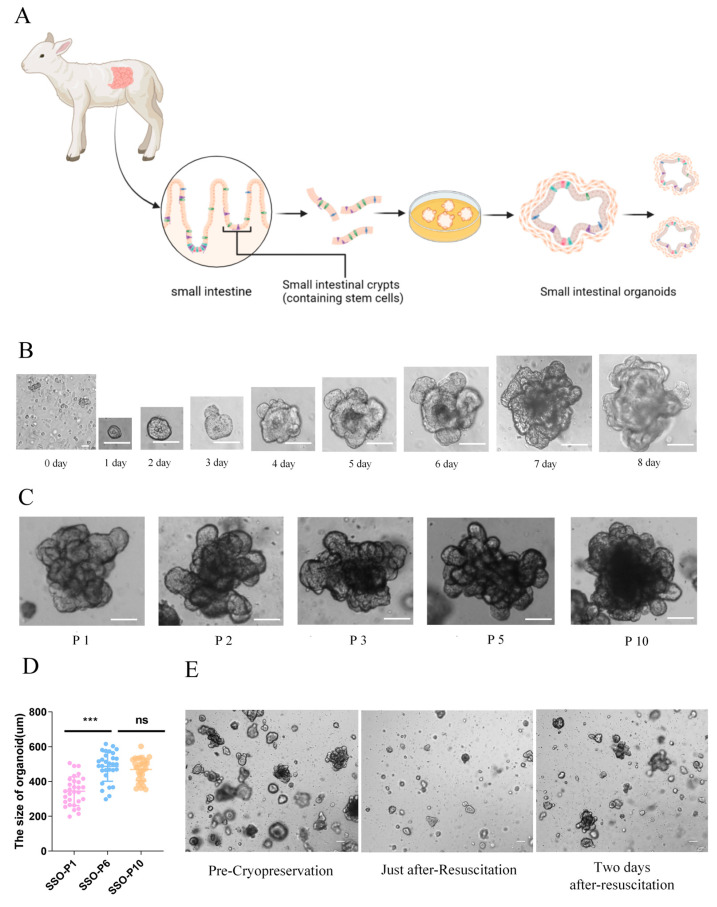
Establishment of the sheep intestinal organoids: (**A**) A schematic illustration of the strategy for developing sheep intestinal organoids, created with BioRender.com. (**B**) Representative images of the same organoid over time derived from isolated intestinal crypts. Three independent experiments were conducted. Scale bars: 100 μm. (**C**) Representative images of the sheep intestinal organoids with different passage numbers indicated below. Three independent experiments were conducted. Scale bars: 100 μm. (**D**) Quantitation of the size of the sheep intestinal organoids at different passages. Three independent experiments were conducted (at least 30 organoids were included in each group). ns, nonsignificant, *** *p* < 0.001. Data were expressed as the mean ± SD by Student’s *t*-test. (**E**) Representative images of organoids prior to cryopreservation, just after resuscitation, and 48 h after resuscitation, respectively. Three independent experiments were conducted. Scale bars: 100 µm.

By far, intestinal organoids derived from numerous species including bovines, pigs, canines, and bats have been successfully established [9,10,12,16,37] and widely applied in studies of nutrient absorption, drug toxicity assessments, microbiota epithelium interaction, etc. However, there are few research reports regarding sheep intestinal organoids. The jejunum, which is located in the middle section of the small intestine, is primarily involved in the absorption of nutrients and has a relatively rich population of stem cells with strong regenerative abilities [16,38,39]. Therefore, jejunum obtained from healthy 8-week-old Hu sheep was used to establish intestinal organoids in vitro. Following the reported Paloma protocol [40], the intestinal crypts were embedded in Matrigel and cultivated in a complete bioGenous^TM^ organoid growth medium (Figure 1A). Within 24 h, the organoids formed closed spherical structures with a central lumen, which branched between day 3 and 5. By day 8, bud-like structures formed from the crypts, indicating that the division and expansion of the crypt domains led to the formation of intestinal organoid structures with a central lumen (Figure 1B). The organoid size increased gradually (Figure 1B) and when the diameter reached approximately 400–500 μm, the organoids were removed from Matrigel, fragmented by pipetting, and passaged in the same culture condition. The organoids had been serially passaged more than 10 times (Figure 1C) and exhibited a rapid increase in volume from passage 1 (P1) to passage 6 (P6), but the volume tended to be stable from passage 6 (P6) to passage 10 (P10), suggesting that organoids experienced significant expansion during the initial culture stages (Figure 1D) and could be maintained through serial passages just as the mouse and human intestinal organoids [6,41]. Moreover, pre-cryopreservation and 48 h after-resuscitation, the organoids exhibited no significant alterations in growth characteristics or morphological phenotypes (Figure 1E). These results demonstrated that sheep intestinal organoids derived from small intestine crypts exhibit a rapid proliferation capacity and can be maintained for the long term without damaging recapitulating capacity.

### 2.2. Cell Lineages Characterization of the Intestinal Organoids

**Figure 2:**
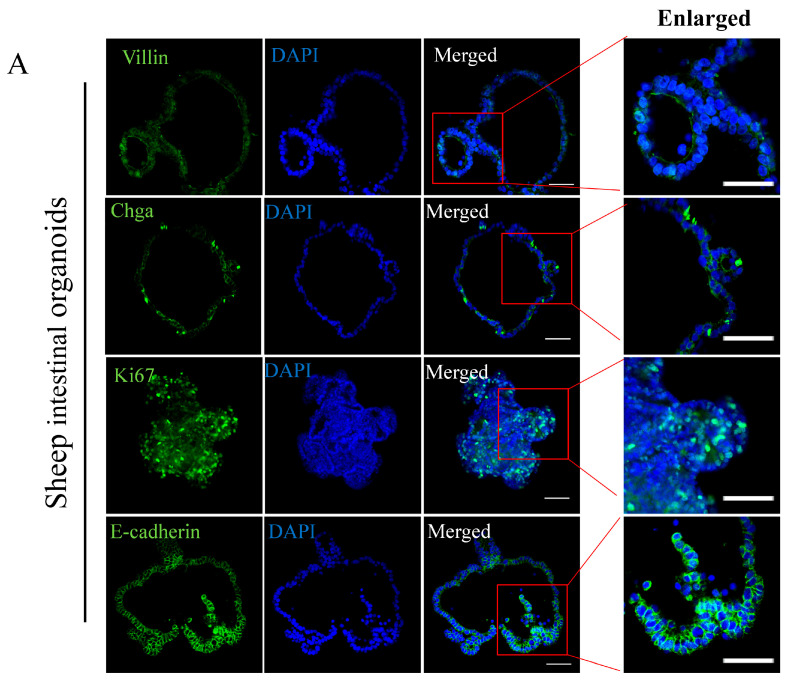

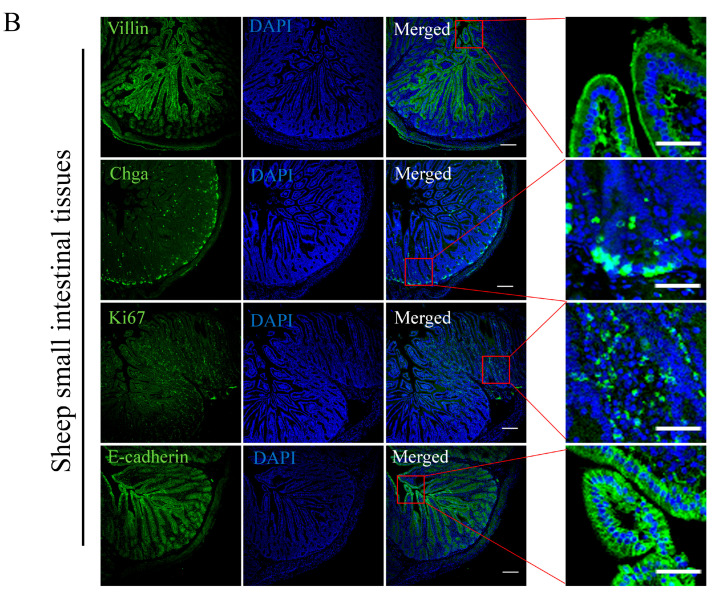
Histological characterization of the intestinal organoids: (**A**) Representative images of immunofluorescence staining in the passage 3 organoids section showing markers for enterocytes (Villin, green), enteroendocrine cells (Chga, green), and intestinal epithelial cells (E-cadherin, green). Transit-amplifying cell markers (Ki67, green) were examined using whole-mount immunostaining. DNA was stained with DAPI (blue). Three independent experiments were conducted. Scale bars: 50 μm, enlarged: 50 μm. (**B**) Immunohistochemistry in sheep intestinal tissue section showing expression of markers for enterocytes (Villin, green), enteroendocrine cells (Chga, green), transit-amplifying cell (Ki67, green), and intestinal epithelial cells (E-cadherin, green). DNA was stained with DAPI (blue). Three independent experiments were conducted. Scale bars: 200 μm, enlarged: 50 μm.

In order to examine the similarity between the sheep intestinal organoids and intestinal tissue, we first performed Hematoxylin and Eosin (H&E) staining and found that sheep intestinal organoids exhibited central intestinal lumen structure surrounded by tightly arranged intestinal epithelial cells (Figure S1A). Immunofluorescence staining showed that the central lumen of the organoids was lined with polarized enterocytes characterized by mature brush borders positive for Villin. Enteroendocrine cells and transit-amplifying cells were scattered throughout the organoid structure shown by positive staining of Chga and Ki67, respectively. Moreover, E-cadherin positively stained a single cell layer of the organoids (Figure 2A). Of note, all these markers for intestinal epithelial cells showed specific staining both in sheep and mouse intestinal tissues (Figure 2B and Figure S1B), validating the specificity of the antibodies. These results collectively confirmed the similarity between organoids and sheep intestinal tissue, and suggested that organoids derived from sheep primary intestine could recapitulate the cell lineage characteristics of the intestinal tissues.

### 2.3. Transcriptional Profiling of the Sheep Intestinal Organoids

**Figure 3:**
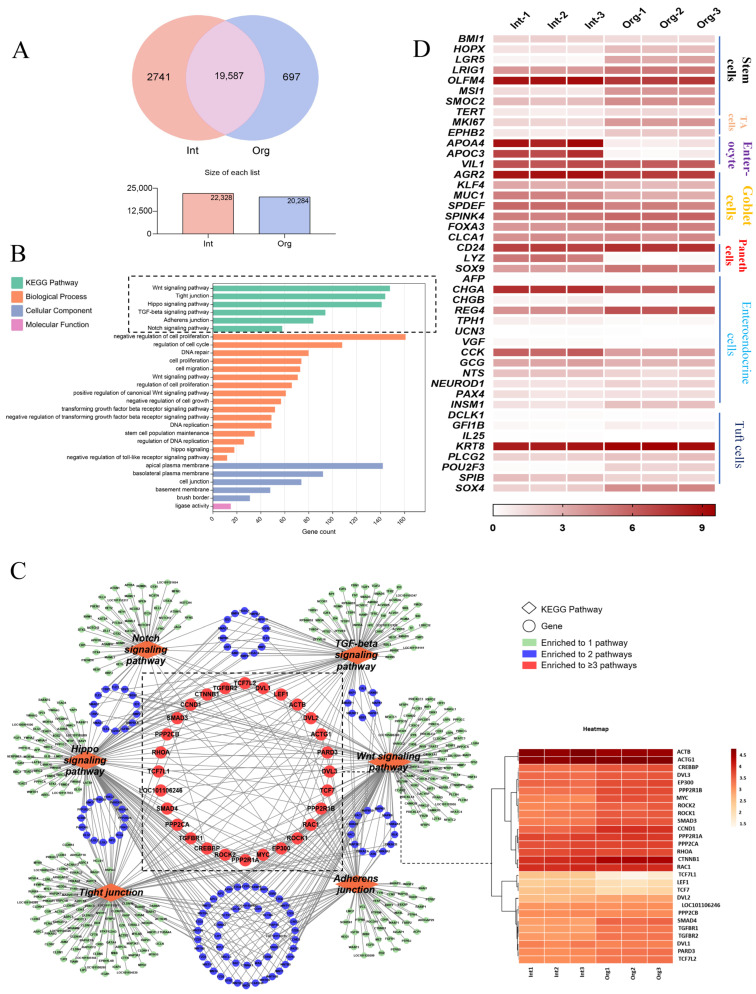
Transcriptional profiling of the sheep intestinal organoids: (**A**) Venn diagram showing co-expressed genes among the organoids and the original intestinal tissue. The red circle represents the number of genes expressed in intestinal tissues, while the blue circle represents the number of genes expressed in the organoids. The intersection of these circles represents the number of genes co-expressed both in the organoids and tissues. (**B**) GO and KEGG enrichment analysis of co-expressed genes in the organoids and tissue. The dotted box highlighting the co-expressed genes are enriched in six signaling pathways essential for intestinal development. (**C**) Network diagram analysis of co-expressed genes enriched in six KEGG signaling pathways. The green circles represent genes enriched in one of the six signaling pathways, the blue circles represent genes enriched in two of the six signaling pathways, and the red circle represents genes enriched in more than three of the six signaling pathways, which are highlighted by the dotted box. Heatmap analysis of co-expressed genes between organoids and tissues, highlighting genes enriched in more than three signaling pathways. (**D**) Heatmap analysis of the expression levels of marker genes associated with stem cells, TA cells, enterocytes, goblet cells, Paneth cells, and tuft cells in the organoids and tissue. Each row represents a gene, and different colors represent expression levels.

In order to confirm the similarity between sheep intestinal organoids and intestinal tissues, transcriptional profiling was conducted using 3 independent samples each for intestinal organoids and intestinal tissue. Venn diagram [42] analysis of the expressed genes revealed that 19,587 genes were co-expressed in both intestinal organoids and tissues (Figure 3A). Subsequently, the co-expressed genes were subjected to functional enrichment analysis using the DAVID functional annotation tool. Gene Ontology (GO) enrichment analysis demonstrated that the biological processes associated with these co-expressed genes were primarily involved in the regulation of the cell cycle, DNA repair, cell proliferation, and cell migration. In terms of cellular components, these co-expressed genes were enriched in the apical plasma membrane, basolateral plasma membrane, basement membrane, cell junction, and brush border (Figure 3B). Meanwhile, Kyoto Encyclopedia of Genes and Genomes (KEGG) analysis found that these co-expressed genes were enriched in six signaling pathways essential for intestinal development, including the Wnt signaling pathway, Hippo signaling pathway, TGF-beta signaling pathway, Notch signaling pathway, Adherens junction, and Tight junction (Figure 3B). Network mapping analysis identified 28 genes that were simultaneously enriched in three of the aforementioned six signaling pathways, while most of the genes were enriched in one or two of the six signaling pathways (Figure 3C). Heatmap analysis of these 28 genes demonstrated that they were highly expressed in both organoids and intestinal tissues (Figure 3C), suggesting that genes involved in intestinal development are consistently active in both organoids and tissues. Based on these results, we continued to examine the expression of genes in specific intestinal epithelial cell subpopulations, including stem cells, enterocytes, goblet cells, tuft cells, and Paneth cells. Heatmap analysis showed that the majority of the genes were consistently expressed both in intestinal tissues and organoids. Notably, the stem cell marker *Lgr5* was more abundantly expressed in intestinal organoids compared to intestinal tissues, indicating a higher proportion of stem cells within the organoids (Figure 3D). However, the expression levels of Paneth cell marker *Lyz* in the organoids were minimal. Consistent with the heatmap obtained from RNA-seq analysis, RT-qPCR analysis revealed that *Lgr5* exhibited higher expression, while expression of *Chga* was down-regulated in the organoid. In contrast, the expressions of *Muc2* and *Villin* did not significantly change (Figure S2), confirming the reliability of the RNA-seq data. Taken together, these results demonstrated that the gene expression properties of sheep intestinal organoids were highly similar to the original tissue and provided strong evidence that the established sheep intestinal organoid faithfully mimics intestinal tissue in vitro.

### 2.4. DON Exposure Inhibits Proliferation of Epithelial Cells and Disrupts Integrity of the Epithelial Cell Barrier in the Organoids

**Figure 4:**
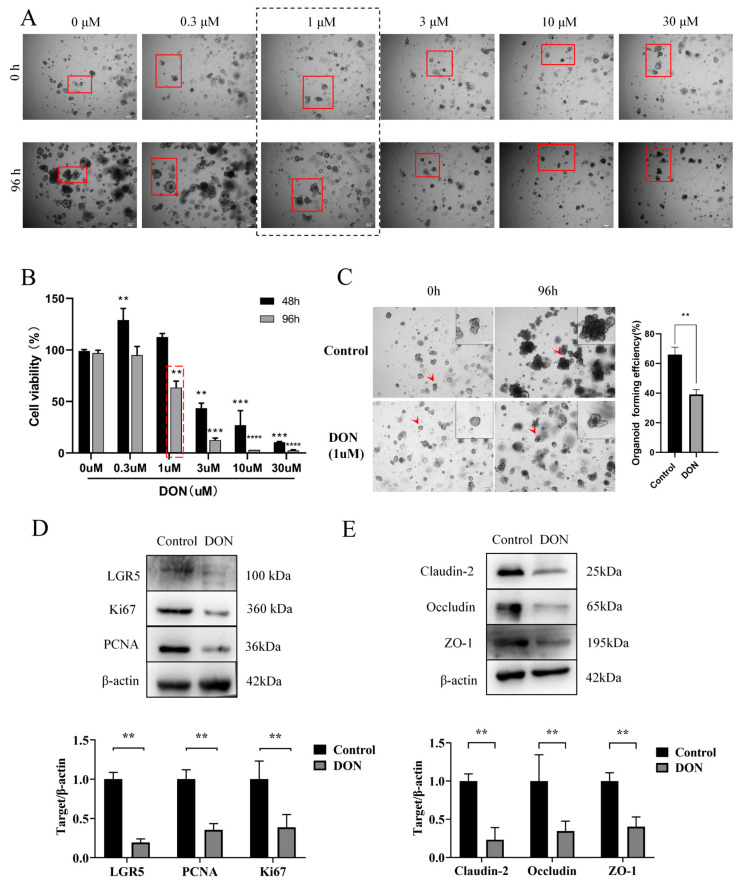
DON exposure inhibits proliferation of epithelial cells and disrupts integrity of the epithelial cell barrier in the organoids: (**A**) Representative images of organoids that were treated with 0, 0.3, 1, 3, 10, 30 μM DON for 0 h and 96 h. The black dotted box highlights the microscopic images of organoids exposed to 1 μM DON for 96 h, while the red square highlights organoids observed at the same location at 0 h and 96 h. Three independent experiments were conducted. Scale bar: 100 μm. (**B**) Cell viability was quantified from three independent experiments using Organoid Viability ATP Assay at 48 and 96 h, respectively. The red dotted box highlights the cell viability of organoids treated with 1 μM DON for 96 h. ** *p* < 0.01, *** *p* < 0.001, **** *p* < 0.0001. Data were expressed as the mean ± SD by two-way ANOVA. (**C**) Representative images of organoids before and after treatment were shown on the left. The inset shows 2× enlarged image of the organoids indicated by red arrows. The histogram on the right quantifies the organoid formation efficiency 96 h after DON treatment. Three independent experiments were conducted (at least 30 organoids were included in each group). ** *p* < 0.01, Data were expressed as the mean ± SD b Student’s *t*-test. Scale bar: 100 μm. (**D**) Expression of PCNA, Ki67, and Lgr5 in control and organoids exposed to DON for 96 h. Representative Western blotting images (**upper** section) and the quantification of WB band intensities from two independent experiments (**lower** section). ** *p* < 0.01, Data were expressed as the mean ± SD by Student’s *t*-test. (**E**) Expression of Occludin, Claudin-2, and ZO-1 in control and organoids exposed to DON for 96 h. Western blotting images (**upper** section) and the quantification of WB band intensities from three independent experiments (**lower** section). ** *p* < 0.01, Data were expressed as the mean ± SD by Student’s *t*-test. DMSO was used as control.

Deoxynivalenol (DON), a mycotoxin produced by *Fusarium* species, is predominantly found as a contaminant in cereal-based foods or feed [43]. DON causes gastrointestinal injury including food refusal, vomiting, diarrhea, and gastrointestinal mucosal damage [44]. However, the underlying mechanisms remain elusive. Therefore, we used the established sheep intestinal organoid (Figure 1, Figure 2 and Figure 3) to investigate the potential impact and mechanism of DON on intestinal epithelia. First of all, five different concentrations of DON (0, 0.3, 1, 3, 10, 30 μM) were added in the culture medium for 48 h and 96 h, respectively, just after passaging the sheep intestinal organoids to determine the optimal treatment concentration. Organoid growth was significantly inhibited after treatment with 1 μM DON for 96 h or 3 μM DON for 48 h. While 0.3 μM of DON had no obvious effect, 10 μM or higher concentrations of DON seriously damaged the organoid structure (Figure 4A and Figure S3A), showing that DON exhibits a dose- and time-dependent toxicity. Consistently, 3 μM of DON significantly reduced cell viability after 48 h exposure, while 1 μM of DON significantly reduced cell viability after 96 h exposure (Figure 4B). Live/dead cell staining further confirmed that after 96 h of DON treatment, the number of live organoids decreased while that of the dead organoids increased with the DON concentration, demonstrating again the dose-dependent cytotoxicity of DON on sheep intestinal organoids (Figure S3B). Since the organoids exhibited a fully budded structure 96 h after passaging, exposure to 1 μM of DON for 96 h was determined as the optimal treatment condition and used in the subsequent experiments. DON treatment significantly reduced organoid volume and damaged organoid forming efficiency (Figure 4C). Given that DON damages intestinal epithelial cells and disrupts the intestinal barrier function [24], we set out to indirectly examine cell proliferation (Figure 4D) and barrier function (Figure 4E) by Western blot analysis. Expression levels of cell proliferation markers PCNA, Ki67, and Lgr5, as well as epithelial cell barrier markers ZO-1, Occludin, and Claudin-2, were all significantly down-regulated in the organoids exposed to DON (Figure 4D,E). These findings collectively suggested that DON exposure inhibited the proliferation of intestinal organoids and disrupted the integrity of the intestinal epithelial cell barrier.

### 2.5. Investigation of the Underlying Mechanism of DON-Induced Intestinal Damage in the Organoids

**Figure 5:**
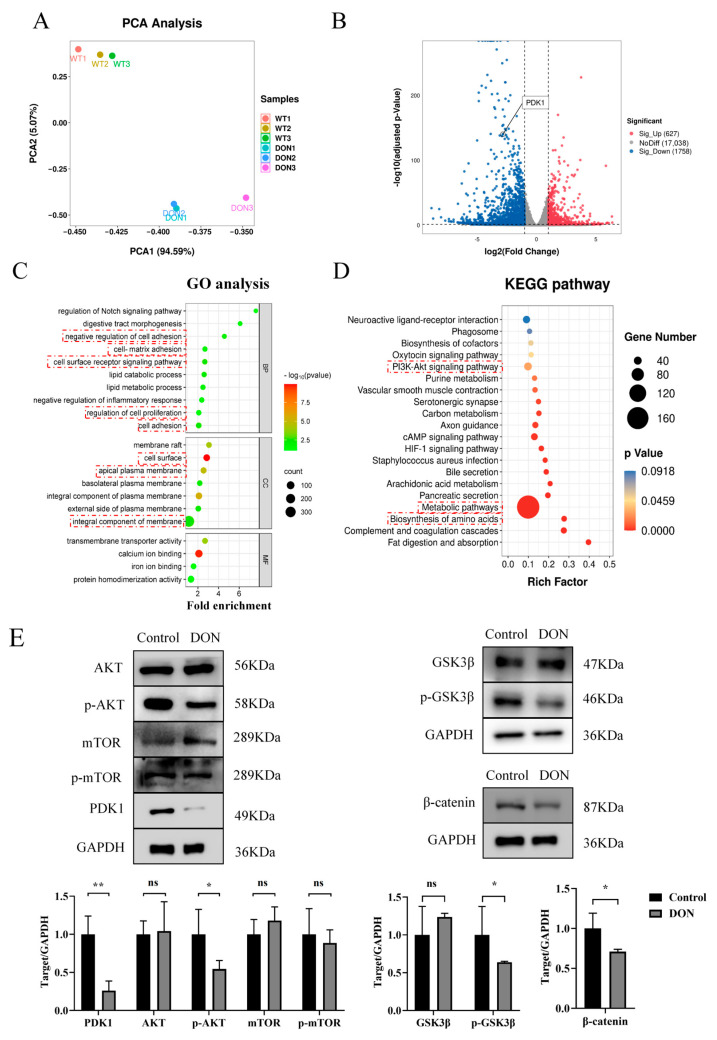
Investigation of the underlying mechanism of DON-induced intestinal damage in the organoids: (**A**) Principal component analysis (PCA) of RNA-seq data from the control and DON-treated organoids. The samples are represented by different colors as indicated on the right. (Control, *n* = 3; DON-treated, *n* = 3). (**B**) Volcano plot showing differentially expressed genes between the control and DON-treated groups. Up-regulated and down-regulated genes are colored in red and blue, respectively. (**C**,**D**) Down-regulated genes significantly enriched for Gene Ontology terms, including Biological Process (BP), Cellular Component (CC), Molecular Function (MF) (**C**), and KEGG pathways (**D**). The biological processes and pathways closely associated with the organoids are highlighted by red dotted box. (**E**) Western blot analysis of key proteins in the PI3K/Akt/GSK3β/β-catenin signaling pathway in the control and DON exposed groups, including PDK1, AKT, GSK3β, and β-catenin. Western blotting images (**upper** section) and the quantification of WB band intensities from three independent experiments (**lower** section). ns, nonsignificant, * *p* < 0.05, Data were expressed as the mean ± SD by Student’s *t*-test.

To address the underlying mechanism of DON-induced intestinal toxicity in-depth, gene expression profiling was performed by RNA sequencing (RNA-seq) on three DON-treated and three control organoid samples. Principal component analysis (PCA) found that the PCA1 axis contributes to the main difference between DON-treated and control groups, accounting for 94.59%. On this axis, transcriptional profiles of DON-treated and control groups were split into two separate clusters showing obviously distinct gene expression patterns (Figure 5A), indicating pronounced transcriptional changes after DON treatment. Further expression analysis identified 2385 differentially expressed genes (adjusted *p*-value < 0.05 and |log_2_Fold Change| > 1) between DON treatment and control groups, including 627 up-regulated genes and 1758 down-regulated genes (Figure 5B, Table S2). In order to gain further insight into the function of the down-regulated genes, the DAVID functional annotation tool was used to perform functional enrichment analysis. GO analysis showed that the down-regulated genes were significantly enriched in processes related to cell adhesion, regulation of cell proliferation, cell surface receptor signaling pathways, and cell–matrix adhesion (Figure 5C). KEGG analysis revealed that the down-regulated genes were significantly enriched in the biosynthesis of amino acids, metabolic pathways, and the PI3K-AKT signaling pathway (Figure 5D). Of note, the PI3K/AKT signaling pathway plays a crucial role in cell growth and survival [45], and the key signaling molecule PDK1 in the PI3K/AKT signaling pathway was significantly down-regulated upon exposure to DON (Figure 5B). These results suggested that DON exposure significantly changed the transcription profile of the organoids, and the PI3K-AKT signaling pathway might serve as a key target for DON.

Since Phosphoinositide-dependent kinase-1 (PDK1) regulates the phosphorylation of its downstream signaling molecules [46], we set out to examine the total protein expression levels and phosphorylation levels of PDK1, Protein kinase B (AKT), Mechanistic target of rapamycin (mTOR), Glycogen synthase kinase 3 beta (GSK3β), and β-catenin by Western blotting. While the total expression levels of PI3K, AKT, mTOR, and GSK3β remained comparable after DON treatment, the phosphorylated form of p-AKT and p-GSK3β, as well as the expression levels of β-catenin and PDK1, was significantly reduced (Figure 5E). These findings suggest that DON exposure has a detrimental effect on intestinal stem cell activity and suppresses cell proliferation by inhibiting the PI3K/AKT/GSK3β/β-catenin signaling pathway and induces intestinal damage thereby.

## 3. Discussion

In recent years, intestinal organoids have garnered significant attention as promising alternatives to traditional animal models [47,48,49], particularly in livestock research involving pigs, chickens, and bovines. However, the cultivation of intestinal organoids is still challenging and influenced by various factors, including donor age and the composition of the culture medium. Initially, we isolated intestinal crypts from adult sheep. However, the crypts had low survival rates, slow proliferation, and failed to develop into complete crypt–villus structures. Only when lambs were used as donors did the obtained organoids exhibit rapid proliferation, capable of long-time culture, cryopreservation, and revival (Figure 1A–E). This is probably due to the abundant stem cell population in young intestines and reminiscent of previous reports in bovine, pig, and mouse [11,50,51,52], highlighting the crucial role of donor age in the successful cultivation of intestinal organoids. In addition, we used the commercial bioGenous^TM^ organoid medium during the culture process, which has a stable composition and does not contain bovine pituitary extract or fetal bovine serum. This medium provided uniform and controllable culture conditions with minimal batch-to-batch variation, which was important for the successful establishment of the sheep intestinal organoids.

The intestinal lumen is lined with a cohesive, polarized simple columnar epithelium that covers upward finger-like protrusions into the lumen called villi and downward invaginations called crypt [53]. Inside the crypts, continuously dividing intestinal stem cells (ISCs) give rise to transit-amplifying (TA) cells that rapidly proliferate and finally differentiate into mature functional intestinal epithelial cells, including enterocytes, goblet cells, enteroendocrine cells, tuft cells, and Paneth cells [3]. Similar to mouse and human intestinal organoids [6], our sheep intestinal organoids robustly express cellular markers for enterocytes (Villin), enteroendocrine cells (Chga), transit-amplifying cells (Ki67), and intestinal epithelial cells (E-cadherin) (Figure 2A), suggesting that the established organoids faithfully mimic the cell lineages of the intestinal epithelium. Intestinal stem cell marker Lgr5 was also detected in the organoids by Western blot (Figure 4D) but not by immunofluorescence staining, probably due to the antibody specificity for WB.

In the complex dynamics of intestinal crypts, a multitude of key signaling pathways collectively regulate intestinal cell fate determination and function [3]. Intriguingly, the transcription profile of the intestinal organoids is highly similar to that of the intestinal tissues, with 19,587 genes co-expressed (Figure 3A). It is worth noting that the genes enriched in signaling pathways essential for intestinal epithelial cell development, such as Wnt, Notch, Hippo, and TGFβ signaling, are also abundantly expressed in the organoid (Figure 3B,C), and are likely responsible for intestinal cellular self-renewal, proliferation, and differentiation [54]. Among them, Wnt signaling primarily drives intestinal stem cell proliferation, Notch signaling is critical in determining the differentiation of intestinal stem cells into absorptive and secretory cells, while the cooperation of these two signaling pathways is crucial for self-renewal and differentiation of intestinal stem cells [55,56]. Additionally, Hippo signaling transduction factors YAP/TAZ promote intestinal stem cell proliferation and intestinal epithelial regeneration, and inhibit Goblet and Paneth cell differentiation [57]. The TGF-β signaling pathway also promotes cell proliferation, differentiation, and growth [58]. These pathways maintain intestinal epithelial homeostasis and are essential for the growth and development of intestinal organoids [59,60]. Therefore, robust expression of the genes enriched in these signaling pathways provided strong evidence that the established sheep intestinal organoids retain key features associated with intestinal tissue and serve as a potential in vitro model for studying intestinal physiology and pathology.

In fact, intestinal organoids have been used as experimental models for studying intestinal diseases, facilitating research on pathogenic bacteria, viruses, nutrient absorption, and interactions among intestinal epithelial cells. Deoxynivalenol (DON), as a feed contaminant, is primarily absorbed in the gastrointestinal tract, enters the liver through the bloodstream, and is eventually excreted in urine by the kidney [61], causing a wide range of enterotoxicity, including disruption of nutrient metabolism, inhibition of cell proliferation, activation of apoptosis, disruption of barrier function, and alteration of the intestinal immune response [29,62,63]. In porcine intestinal organoids, treatment with 250 or 500 ng/mL (approximately 1.1 μM or 2.2 μM) of DON for 24 h suppressed organoid expansion and significantly decreased budding efficiency [35]. Similarly, consistent with porcine studies, DON treatment also significantly reduced the formation efficiency and cell viability of sheep intestinal organoids (Figure 4A–C). Given that DON exposure damages not only the intestinal epithelium but also the intestinal stem cells (ISCs) [64,65,66,67], the observed effect is likely due to impaired intestinal epithelial cell proliferation and intestinal stem cell expansion. Indeed, the expression of genes related to cell proliferation and barrier function was remarkably decreased after DON treatment (Figure 4D,E). Recently, it has been reported in cshicken intestinal organoids that exposure to 2 μg/mL (approximately 9.5 μM) of DON for 24 h damages the barrier function of the organoids, which resulted in increased epithelial barrier permeability [68]. Research on mouse intestinal organoids showed that exposure to 1 μM DON, both basolateral exposure and luminal exposure, compromised intestinal barrier function and stem cell viability [36]. Therefore, although at the same order of magnitude, the sensitivity of sheep intestinal organoids to DON is similar to that of mouse intestinal organoids but more sensitive than that of porcine and chicken intestinal organoids.

Our in-depth analysis of transcriptome data identified PI3K/AKT as a key signaling pathway impaired by DON treatment in the sheep intestinal organoids (Figure 5A–D), which is well known to play important roles in various cellular processes such as cell proliferation, cell-cycle progression, apoptosis, protein synthesis, and glucose metabolism [69]. In this signaling pathway, AKT is phosphorylated and activated by PI3K, then regulates a number of downstream effectors, including GSK3β and mTORC1 [70]. GSK3β is phosphorylated by p-AKT, and the p-GSK3β then activates the β-catenin pathway [71]. Both the GSK3β and β-catenin are key regulators of the Wnt/β-catenin signaling pathway, which is essential for various fundamental cellular processes including proliferation, differentiation, migration, self-renewal, and apoptosis [72]. Although having little effect on mTOR, DON exposure significantly reduced p-AKT, p-GSK3β, β-catenin, and PDK1 levels (Figure 5E). It has been shown in tumor cells that inhibition of p-AKT, p-GSK3β, as well as β-catenin, greatly reduce cell proliferation, migration, and invasion [73], supporting the notion that the reduced formation efficiency and cell viability of sheep intestinal organoids after DON treatment is due to impaired PI3K/AKT/GSK3β and Wnt/β-catenin signaling pathways, at least partially. Moreover, previous studies both in mouse and porcine reported that DON exposure suppresses LGR5 and β-catenin expression, leading to reduced activity of intestinal organoids [35,36], which is also in line with our findings (Figure 4D and Figure 5E). Therefore, it is likely that DON induces intestinal toxicity by inhibiting the PI3K/AKT/GSK3β/β-catenin signaling pathways, which might be a shared mechanism in mammalian species.

## 4. Materials and Methods

### 4.1. Isolation of Crypts from Sheep Intestine and the Subsequent In Vitro Culture

All animal procedures were conducted in accordance with the institutional guidelines and regulations for animal care and use. The study was approved by the Institutional Animal Care and Use Committee of Inner Mongolia University. Briefly, 8-week-old lambs of Hu sheep [74] were euthanized, and a 5–10 cm segment of the jejunum from the middle section of the small intestine was isolated immediately. The crypts’ isolation was conducted according to a previous report [75]. Briefly, the sheep intestinal tissues were cut longitudinally and washed in cold PBS 3–4 times to remove the contaminant and feces. Subsequently, adipose and vascular tissues were removed using a scalpel. The remaining tissues were cut into small pieces approximately 5 mm in diameter and incubated in 5 mM EDTA (Coolaber, Beijing, China) for 30 min. After removal of EDTA, the small pieces were vigorously suspended using a 5 mL pipette with cold PBS. The supernatant, which was enriched with intestinal crypts, was passed through a 70 μm cell strainer (Biosharp, Anhui, China) and centrifuged at 300× *g* for 3 min. The crypts were then embedded in growth factor-reduced Matrigel (bioGenous^TM^, Hangzhou, China) and seeded in the middle of a 24-well plate. Once polymerization was complete, the organoid expansion medium (bioGenous^TM^, Hangzhou, China) was added. The organoid maintenance medium (bioGenous^TM^, Hangzhou, China) was refreshed every 3 days.

### 4.2. Passaging and Cryopreservation of the Sheep Intestinal Organoids

Sheep intestinal organoids were passaged approximately every 4–5 days after maturation. Briefly, the medium was gently aspirated, and the organoid Matrigel mixed suspension was rinsed with ice-cold PBS. The suspension was then pipetted vigorously to disrupt the Matrigel and transferred into a 1.5 mL conical tube. After centrifugation at 300× *g* for 3 min at room temperature, the pellet was resuspended in a 10× volume dissociation solution (bioGenous^TM^, Hangzhou, China) in a 1.5 mL conical tube, followed by 3 min incubation in an incubator to harvest the organoids. The organoids were passaged at a 1:3 to 1:4 split ratios, embedded in new Matrigel matrix, and cultured in a maintenance medium (bioGenous^TM^, Hangzhou, China). To enable long-term storage, the organoids were cryopreserved in a serum-free cryopreservation medium (bioGenous^TM^, Hangzhou, China) and subsequently placed in −80 °C or liquid nitrogen. When required, the cryopreserved organoids were recovered effectively using the organoid expansion medium (bioGenous^TM^, Hangzhou, China).

### 4.3. Total RNA Extraction and RT-qPCR

RT-qPCR assay was performed as previously described [76]. Briefly, total RNAs from cultured organoids were extracted with RNAiso Plus (Takara, Osaka, Japan) according to the manufacturer’s instructions and the concentration was determined by a Nanodrop ND-1000 spectrophotometer. The RNA was transcribed into corresponding deoxyribonucleic acid (cDNA) using the PrimeScript^TM^ RT Reagent Kit with gDNA Eraser (Takara, Osaka, Japan). The reaction mixture consisting of 1 μL cDNA, 6.25 μL TB Green Premix Taq II (2×), 0.4 μL of each primer, and 4.45 μL ddH_2_O was incubated in LightCycler480 real-time PCR system (Roche, Basel, Switzerland). The PCR protocol used was 95 °C for 30 s; (2×), 0.4 μL 40 cycles of 95 °C for 5 s; and 60 °C for 20 s. All amplifications were performed in technical duplicate and biological triplicate. The RT-qPCR data were analyzed using LightCycler 96 SW 1.1 software and relative expression levels of the target genes were calculated by the 2^−ΔΔCT^ method. The primer sequences are listed in Table S1.

### 4.4. Hematoxylin & Eosin Staining and Immunohistochemical Staining

Sheep intestinal organoids were released from the Matrigel and incubated with cell recovery solution (bioGenous^TM^, Hangzhou, China) on a horizontal shaker at 4 °C (60 rpm) for 30 min. Organoids were centrifuged at 300× *g* for 5 min. The medium was removed, and the organoids were resuspended in 4% paraformaldehyde (PFA) (Sigma-Aldrich, St. Louis MO, USA) and incubated for 1 h at 4 °C. The fixed sheep intestinal organoids were then embedded in 3% agarose. Subsequently, the organoids were dehydrated and embedded in paraffin wax. Hematoxylin & Eosin (H&E) staining and immunohistochemical staining were performed on 5 µm sections of paraffin-embedded organoids. Paraffin-embedded sections of organoids were dewaxed, and antigen retrieval was performed by pretreating in boiling citrate buffer pH 6 (50×, Solarbio, Beijing, China). Subsequently, slides were incubated in a blocking buffer consisting of 0.1% Triton X-100 (Sigma-Aldrich, St. Louis, MO, USA) and 5% bovine serum albumin (BSA; Sigma-Aldrich, St. Louis, MO, USA) in PBS for 1 h at room temperature. Slides were incubated overnight at 4 °C with the following primary antibodies: anti-Chga (Proteintech, Wuhan, China), anti-E-cadherin (Cell Signaling Technology, Danvers, MA, USA), anti-Villin (Proteintech, Wuhan, China), and anti-Olfm4 (Cell Signaling Technology, Danvers, MA, USA). After washing, the slides were incubated with anti-mouse and anti-rabbit secondary antibodies coupled to Alexa Fluor-488 (Cell Signaling Technology, Danvers, MA, USA) or Alexa Fluor-594 (Cell Signaling Technology, Danvers, MA, USA) for 1 h at room temperature. Diamidino-2-phenylindole (DAPI) (biosharp, Hefei, China) was used to counterstain the DNA. All images were obtained with a confocal laser-scanning microscope (Zeiss, Oberkochen, Germany).

### 4.5. Immunofluorescence Staining

Following Hans Clevers’ protocol [77], sheep intestinal organoids were released from the Matrigel and incubated with cell recovery solution (bioGenous^TM^, Hangzhou, China) on a horizontal shaker at 4 °C (60 rpm) for 30 min. Organoids were centrifuged at 300× *g* for 5 min. The pellet of organoids was gently resuspended in 4% PFA and incubated at 4 °C for 1 h. Subsequently, the organoids were washed 3 times with PBS and permeabilized with 0.25% Triton X-100 for 1 h at room temperature. Then, the organoids were blocked with 5% BSA solution for 2 h at room temperature. Organoids were incubated overnight at 4 °C with primary antibody for anti-Ki67 (PTM-BIO, Hangzhou, China). After washing 3 times, the samples were incubated with fluorescein-labeled secondary antibody for Alexa Fluor-488 (Cell Signaling Technology, Danvers, MA, USA) and Diamidino-2-phenylindole (DAPI) for 1 h at room temperature the next day. The images were captured using confocal microscopes (Zeiss, Oberkochen, Germany).

### 4.6. DON Exposure of the Sheep Intestinal Organoids

Before exposure to DON, sheep intestinal organoids were digested into small clumps or single cells with dissociation solution. The cells were cultured for 24 h to form spheroids, and subsequently treated with different concentrations of DON for 48 h or 96 h. Different concentrations of DON were applied based on previous reports [35,36]. Organoid growth was examined continuously under a microscope, and cell viability was measured by 3D Cell Viability Assay (bioGenous^TM^, Hangzhou, China) according to the manufacturer’s instructions. Prior to the assay, organoids were seeded in 96-well plates and exposed to DON for 48 h and 96 h. Subsequently, 50 μL of 3D Cell Viability solution was added to each well, and the organoids were incubated in dark for 3 h. The absorbance intensity was then measured at 560 nm using a microplate reader (BioTek, Winooski, VT, USA).

### 4.7. Extraction of Total Proteins and Western Blotting

Extraction of total proteins and Western blotting were performed according to a previous report [78]. Briefly, tissues and organoids were lysed in RIPA lysis buffer (Beyotime, Shanghai, China) with protease inhibitor (Beyotime, Shanghai, China) and phosphatase inhibitor (Beyotime, Shanghai, China). Total protein concentration was measured by the Bradford assay and protein samples were separated by 12% sodium dodecyl sulfate polyacrylamide gel electrophoresis, and transferred to a polyvinylidene fluoride membrane (Millipore, Burlington MA, USA). The membrane was blocked with 5% skimmed milk at room temperature (RT) for 1 h, probed with primary antibodies overnight, and subsequently incubated with HRP-conjugated secondary antibodies. The antibodies used in this study are listed as follows. Primary antibodies: anti-Claudin-2 (Affinity Biosciences, Changzhou, Jiangsu, China), anti-LGR5 (Affinity Biosciences, Jiangsu, China), anti-Occludin (Affinity Biosciences, Jiangsu, China), anti-ZO-1 (Affinity Biosciences, Jiangsu, China), anti-PDK1 (Huaan biotechnology, Hangzhou, China), anti-Phospho-mTOR (Huaan biotechnology, Hangzhou, China), anti-mTOR (Huaan biotechnology, Hangzhou, China), anti-AKT (Proteintech, Wuhan, China), anti-Phospho-AKT (Proteintech, Wuhan, China), anti-GSK3β (Cell Signaling Technology Technology, Danvers, MA, USA), anti-Phospho-GSK3β (Absin, Shanghai, China), anti-β-catenin (Proteintech, Wuhan, China), anti-β-actin (Proteintech, Wuhan, China), and anti-GAPDH (Proteintech, Wuhan, China). Secondary antibodies: goat anti-mouse IgG-HRP (Abcam, Cambridge, UK) and goat anti-rabbit IgG-HRP (Abcam, Cambridge, UK).

### 4.8. RNA-Seq and Data Analysis

Total RNA was extracted from intestinal tissues (int), intestinal organoids (org), and DON-treated (DON) organoids using RNAiso Plus according to the manufacturer’s instructions (Takara, Osaka, Japan ). Sequencing libraries were constructed using VAHTS Universal V6 RNA-seq Library Prep Kit for Illumina (Vazyme, Nanjing, China). Three biological replicates were performed in each group. Agilent 2100 Bioanalyzer was used for quality control of the sequencing libraries. The libraries were sequenced paired-end 2 × 150 bp using NovaSeq 6000 and each library was sequenced to obtain at least 6 Gb data (Annoroad, Beijing, China). Then, raw reads were subjected to quality filtering and adaptor trimming using fastq (version 0.3.2). High-quality reads were subsequently aligned against the genome sequence of the sheep genome (oviAri4) using hisat2 (version 2.2.1). Raw counts per gene were obtained using Feature Counts. Similarity expression analysis was performed with the R package DESeq2 (adjusted *p*-value < 0.05 and |log_2_Fold Change| > 1). DAVID was used to conduct Gene Ontology (GO) and Kyoto Encyclopedia of Genes and Genomes (KEGG) analysis for similarly expressed genes (https://david.ncifcrf.gov/ (accessed on 18 September 2023)).

### 4.9. Statistical Analysis

Quantitative data were expressed as the mean ± SD. Student’s *t*-test or two-way ANOVA analyses were used to compare the difference between two groups or multiple groups as indicated in the figure legends. To compare the differences in quantitative data between groups, the normal distribution of data was verified, and statistical analysis was carried out by analysis of variance (ANOVA). GraphPad Prism 7 software (GraphPad Software, La Jolla, CA, USA) was used for statistical analysis. *p* < 0.05 was used to determine significant difference. * Indicates *p* < 0.05, ** indicates *p* < 0.01 and *** indicates *p* < 0.001, **** indicates *p* < 0.0001. The size of organoids was quantified with ImageJ Version 1.54m. Each experiment was repeated independently at least three times.

## 5. Conclusions

In this study, we successfully developed sheep intestinal organoids with the capabilities of long-term passaging, cryopreservation, and resuscitation. Histological and transcriptome analyses revealed a high degree of cell lineage and gene expression similarity between sheep intestinal organoids and original intestinal tissues, demonstrating the successful establishment of the sheep intestinal organoids. Further application in DON-induced intestinal toxicity revealed that DON inhibits organoid proliferation through the PI3K/AKT/GSK3β/β-catenin signaling pathway, thereby impairing organoid proliferation and barrier integrity, and highlighting that sheep intestinal organoids potentially offer a valuable in vitro model for intestinal research.

## Data Availability

The RNA-seq data were deposited to the NCBI SRA database (PRTNA1063240, PRJNA1063360). All data associated with this study are available by contacting the corresponding authors with a request.

## Supplementary Materials

The supporting information can be downloaded at: https://www.mdpi.com/article/10.3390/ijms26030955/s1.
