# Supplementary material for: Development of Sheep Intestinal Organoids for Studying Deoxynivalenol-Induced Toxicity
Source: Int J Mol Sci. 2025 Jan 23;26(3):955. doi: 10.3390/ijms26030955 (PMC11816529; doi:10.3390/ijms26030955)
Supplement: Supplementary file 1 [file ijms-26-00955-s001.zip › Supplementary Materials.pdf]

Supplementary Materials

Article

Development of Sheep Intestinal Organoids for Studying Deoxynivalenol-Induced Toxicity

Hongyu Wang [1,†], Xige He [1,†], Miaomiao Zhang [1], Na Fan [1], Zongxuan Yang [1], Ting Shen [1,2], Jiaojiao Guo [1,2], Yongli Song [1,2], Guifang Cao [1,2,3], Yongbin Liu [1,2] and Xihe Li [1,2,3,*] and Buhe Nashun [1,2,*]

[1] Inner Mongolia Key Laboratory for Molecular Regulation of the Cell, Inner Mongolia University, Hohhot 010070, China; 22408045@mail.imu.edu.cn (H.W.); 131994998@imu.edu.cn (X.H.); 32308061@mail.imu.edu.cn (M.Z.); 22008037@mail.imu.edu.cn (N.F.); 22108042@mail.imu.edu.cn (Z.Y.); 0222122876@mail.imu.edu.cn (T.S.); gijlife@imu.edu.cn (J.G.); ylsong@imu.edu.cn (Y.S.); guifangcao@126.com (G.C.); ybliu@imu.edu.cn (Y.L.)

[2] State Key Laboratory of Reproductive Regulation and Breeding of Grassland Livestock, School of Life Sciences, Inner Mongolia University, Hohhot 010040, China

[3] Inner Mongolia Saikexing Institute of Breeding and Reproductive Biotechnology in Domestic Animals, Hohhot 011517, China

* Correspondence: lixh@imu.edu.cn (X.L.); bnashun@imu.edu.cn (B.N.); Tel.: +86-(0)471-4996885 (B.N.)

† These authors contributed equally to this work.

Figure S1

A

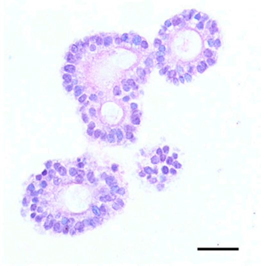

B

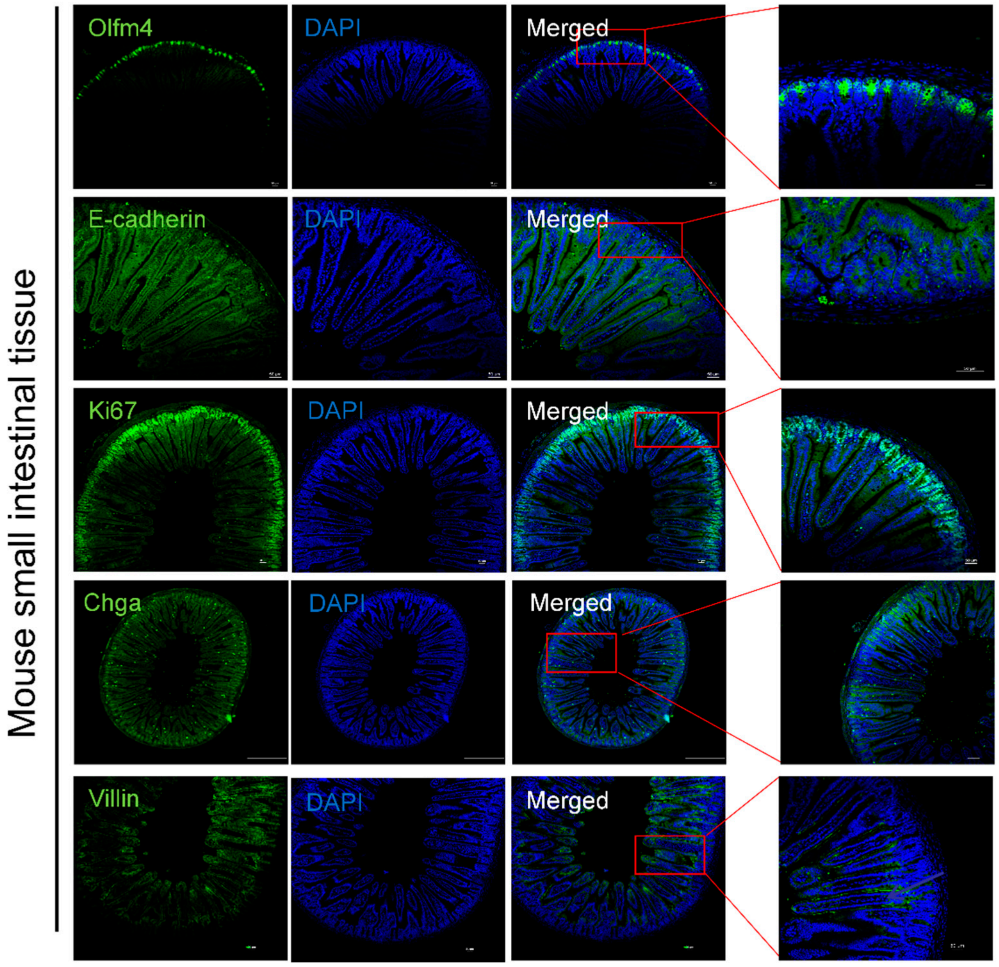

Figure S1. Representative Images of Sheep Intestinal Organoids and Validation of Antibody Specificity in Mouse Intestinal Tissues

(A) Representative images of sheep intestinal organoid sections stained with Hematoxylin and Eosin (H&E). Scale bars: 100 μm. **(B)** Validation of antibody specificity in intestinal tissues of mice. Olfm4 (intestinal stem cell marker), E-cadherin (intestinal epithelial cell marker), Ki67 (transit-amplifying cell marker), Chga

(enteroendocrine cell marker) and Villin (enterocytes marker) were all positively stained in intestinal tissues of mice. Scale bar: 50 μm.

Figure S2

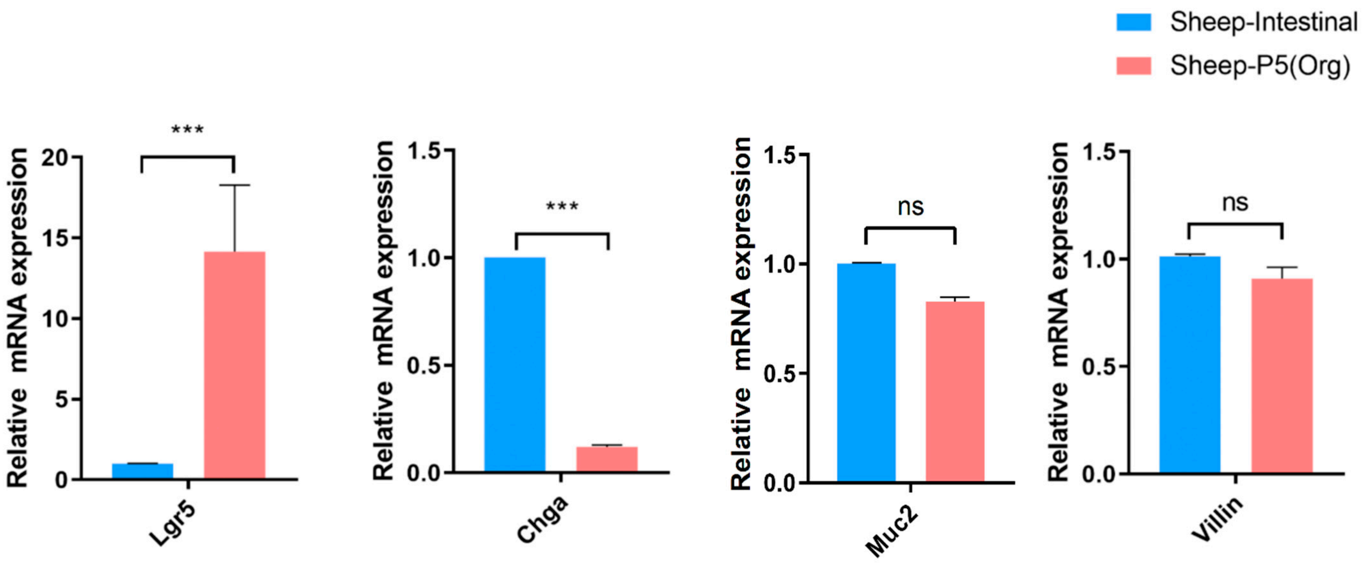

Figure S2. Expression of Cell Marker Genes in Sheep Intestinal Organoids and Original Intestinal Tissues by RT-qPCR

Expression of cell marker genes (*Lgr5, Chga, Muc2, Villin*) by RT-qPCR assay in the sheep intestinal organoids and original intestinal tissues. ns, nonsignificant, ***$p < 0.001$. Data were displayed as the mean ± SD by Student's t-test. Three independent experiments were performed.

Figure S3

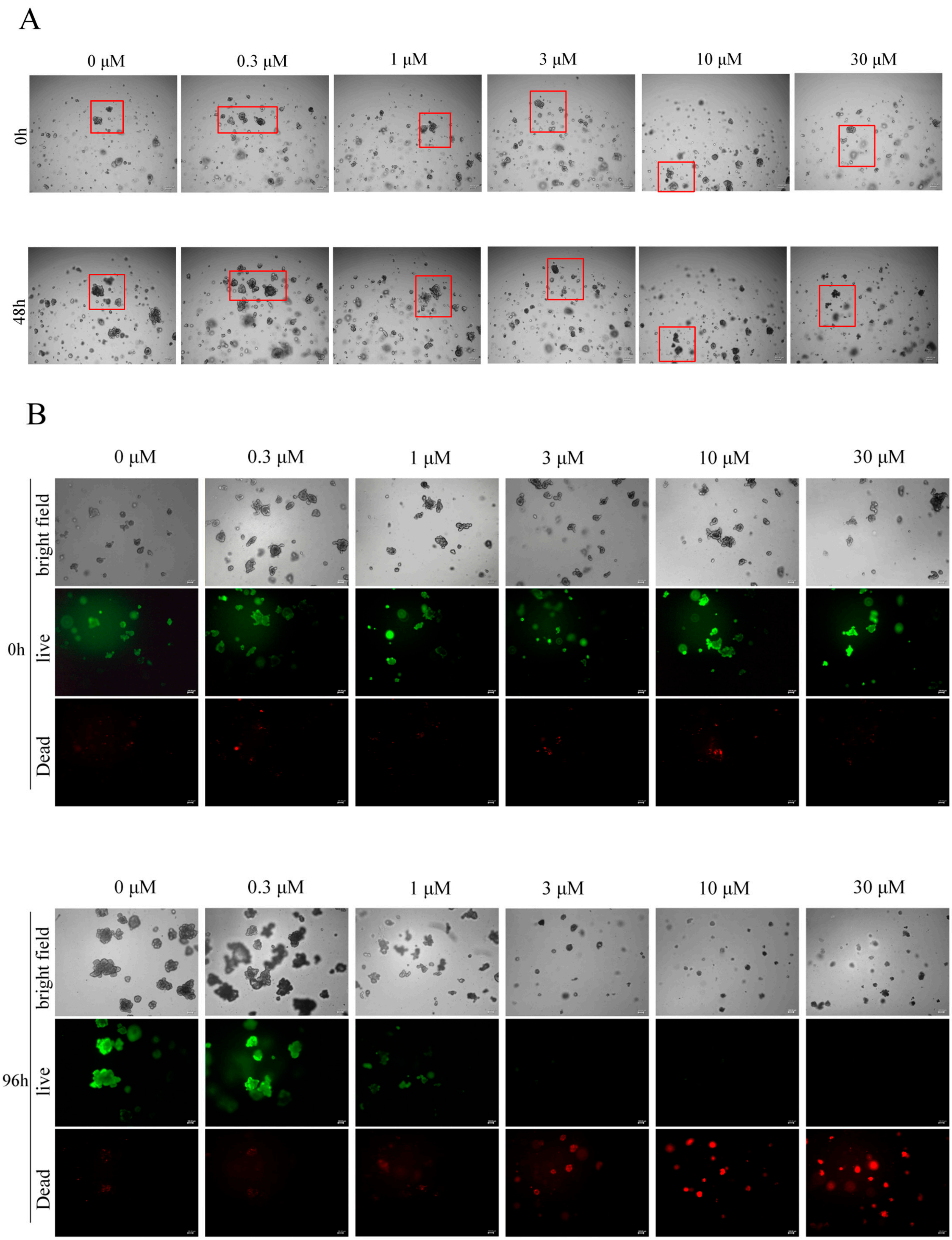
A
0 μM
0.3 μM
1 μM
3 μM
10 μM
30 μM
0h
48h
B
0 μM
0.3 μM
1 μM
3 μM
10 μM
30 μM
bright field
live
Dead
0h
0 μM
0.3 μM
1 μM
3 μM
10 μM
30 μM
bright field
live
Dead
96h

Figure S3. Representative Images of Intestinal Organoids Treated with Various Concentrations of DON for 48 h and Live/Dead Cell Staining of Organoids Treated for 0 h and 96 h

(A) Representative images of organoids that were treated with 0, 0.3, 1, 3, 10, 30 μM DON for 0 h and 48 h. Three independent experiments were conducted. Scale bar: 100 μm. **(B)** Representative images of live/dead cell staining of the intestinal organoids treated with DON for 0 h and 96 h (green fluorescence indicating live cells stained with Calcein-AM and red fluorescence indicating dead cells stained with propidium iodide (PI)). Three independent experiments were conducted. Scale bar: 100 μm.

Supplementary Table S1: Primers for Quantitative RT-qPCR

Supplementary Table S2: Differentially Expressed Genes Between Control and DON Treatment in Organoids
